# Mediators and Moderators in the Co-Occurring Anxiety and Alcohol Use Relationship: Protocol for a Systematic Review and Meta-Analysis

**DOI:** 10.2196/48875

**Published:** 2023-07-13

**Authors:** Tara Guckel, Katrina Prior, Nicola Clare Newton, Lexine Ann Stapinski

**Affiliations:** 1 The Matilda Centre for Research in Mental Health and Substance Use University of Sydney Camperdown Australia

**Keywords:** alcohol use, alcohol, anxiety, comorbidity, drinking, electronic databases, mediator, meta-analysis, moderator, prevention, systematic review

## Abstract

**Background:**

Anxiety and alcohol use commonly co-occur. Previous research has demonstrated the bidirectional and mutually reinforcing nature of this relationship, with an increasing body of research investigating the mediating and moderating mechanisms driving this association. Within the existing mediation and moderation research, however, there have been contrasting findings and, at times, null results among some population groups.

**Objective:**

This protocol outlines a systematic review and meta-analysis aiming to synthesize and clarify mediators and moderators in the anxiety-alcohol and alcohol-anxiety relationships.

**Methods:**

Systematic searches will be conducted in the electronic databases Medline, PsycINFO, Embase, Cochrane Central Register of Controlled Trials, Scopus, and Web of Science to identify studies that investigated mediators and moderators of the relationship between anxiety and alcohol use, including clinical and subclinical levels. Studies that look at the relationship between anxiety and alcohol use outcomes, as well as alcohol use and anxiety outcomes, will be included in order to capture an in-depth understanding of the mechanisms driving the association in either direction. No limits will be placed on study year or study language. Included study designs will be observational studies, including cohort, cross-sectional, and longitudinal studies, and secondary analyses of randomized controlled trials reporting quantitative results. Selected studies will also have their reference lists hand-searched for other relevant papers. Study quality will be assessed with the Joanna Briggs Institute Checklists for Analytical Cross-Sectional Studies and Cohort Studies. Mediators and moderators will be narratively synthesized in line with the biopsychosocial framework, where results will be grouped into biological, psychological, and social or environmental factors. If the data are sufficiently homogeneous, a meta-analysis will be conducted with mediation and moderation analyses synthesized separately. The Grading of Recommendations, Assessment, Development, and Evaluations (GRADE) framework will also be used to assess the strength of cumulative evidence.

**Results:**

Electronic database searches were conducted in September 2022. After duplicates were removed, a total of 7330 titles and abstracts were screened. Full-text reviewing is currently under way, with the results expected to be available by the end of 2023.

**Conclusions:**

Given the significant individual and societal impacts of co-occurring anxiety and alcohol use, this review will help clarify mechanisms linking these two concerns. Identified mechanisms, where possible, can then be targeted in prevention, early intervention, and treatment approaches to improve the outcomes for individuals experiencing co-occurring anxiety and alcohol use.

**Trial Registration:**

PROSPERO CRD42023358402; https://tinyurl.com/2m2e3enp

**International Registered Report Identifier (IRRID):**

DERR1-10.2196/48875

## Introduction

Globally, anxiety disorders are the most common psychiatric disorders, with an estimated 301 million individuals experiencing one or more anxiety disorders in their lifetime [1]. Alcohol use is also highly prevalent, with an estimated 32.5% of people aged 15 years or older globally being current drinkers [2]. Among drinkers, high rates of risky drinking and alcohol use disorders are seen. A large nationally representative survey of 36,309 adults in the United States found 12.9% of individuals drink at high-risk levels, defined as 4+ standard drinks for women and 5+ standard drinks for men, at least weekly [3]. Additionally, among those who drink, 17.5% experienced an alcohol use disorder in the past year [3]. Comorbidity between mental health and substance use concerns is common, most notably between anxiety and alcohol use. Indeed, among individuals with an anxiety disorder, it is estimated that 20%-40% will also experience an alcohol use disorder in their lifetime [4]. When anxiety and alcohol use co-occur, symptom severity can be worse, and treatment adherence and outcomes are typically poorer compared to individuals with an anxiety or alcohol disorder alone [5,6].

Several theories have been proposed to explain the comorbid, bidirectional relationship between anxiety and alcohol use. Arguably, the most common is the self-medication model [7,8] which suggests individuals drink alcohol to alleviate symptoms of anxiety or general negative affect, in turn leading to a reliance on alcohol over time and the development of a subsequent alcohol use disorder. The use of alcohol for self-medication also aligns with other theories, including the tension-reduction theory [9] and the stress response dampening model [10]. Conversely, there is the opposite causal explanation that alcohol misuse and alcohol use disorders promote anxiety disorders, known as the substance-induced anxiety model or kindling or stress hypothesis [6,11,12]. Following prolonged periods of heavy alcohol consumption, withdrawal or cessation can lead to adaptations in the brain and wider nervous system, which can induce or worsen anxiety [6,11]. In addition to evidence of causal, bidirectional pathways between anxiety and alcohol, there is also support for a mutual-maintenance model also referred to as the “vicious cycle of comorbidity” [13]. This model demonstrates how the biopsychosocial outcomes of one disorder (eg, anxiety) will often serve to maintain or exacerbate the other disorder (eg, alcohol use disorder). An example of such a relationship can be seen when an individual with social anxiety who is self-medicating with alcohol develops a reliance on alcohol and over time experiences alcohol-related consequences like decreased productivity, interpersonal issues, and anxiety-induced withdrawal. These consequences exacerbate the initial anxiety and result in further drinking, thus maintaining the problematic feed-forward cycle [6,13]. Together, these theoretical frameworks suggest a complex bidirectional relationship between anxiety and alcohol use, with longitudinal and prospective studies confirming that experiencing an anxiety disorder or an alcohol use disorder can increase the risk of developing the other [14,15].

Previous critical and systematic reviews have established a strong body of evidence for a relationship between anxiety and alcohol use [12,16]. These reviews have typically focused on the temporal sequencing of the anxiety-alcohol relationship [16,17]; comorbidity between a specific anxiety disorder, for example, social anxiety disorder, and alcohol use [5,18-21]; the treatment of comorbid anxiety and alcohol use disorders [22-25]; or all of the above [12,26]. Yet, despite substantial existing research, the specific mechanisms driving anxiety and alcohol comorbidity are still poorly understood. The proposed systematic review will be the first to synthesize and clarify the specific mediators and moderators driving the relationship between anxiety and alcohol use.

**Figure 1:**
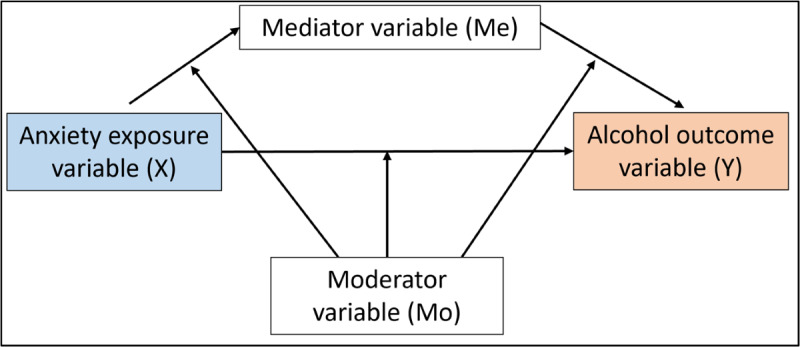
Diagram demonstrating the possible mediator (Me) and moderator (Mo) pathways between the exposure variable anxiety (X) and outcome variable alcohol (Y).

**Figure 2:**
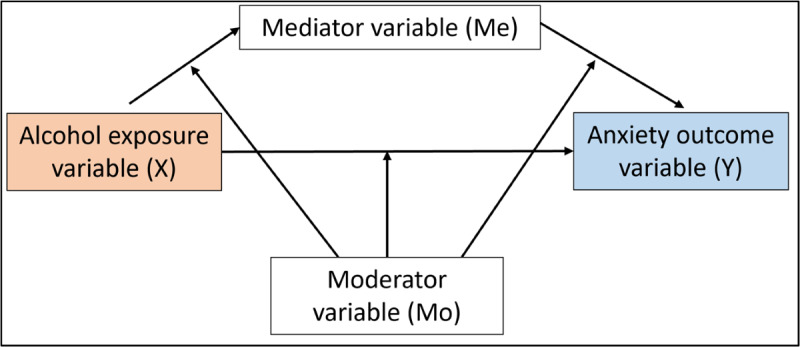
Diagram demonstrating the possible mediator (Me) and moderator (Mo) pathways between the exposure variable alcohol (X) and outcome variable anxiety (Y).

Given the bidirectional nature of comorbid anxiety and alcohol use, the planned review will investigate the relationship in both directions. Figures 1 and 2 are path diagrams visually demonstrating the relationships that will be examined. Figure 1 illustrates anxiety as the exposure/predictor variable (X) and alcohol use as the outcome variable (Y), while Figure 2 illustrates the relationship in the opposite direction, where alcohol use is the exposure/predictor (X) and anxiety is the outcome (Y). As illustrated in Figures 1 and 2, a mediator or moderator is a third variable that affects the relationship between the exposure and outcome of interest [27]. Investigating mediators is warranted to better understand causal mechanisms that account for a relationship between X and Y [28]. In short, by identifying mediators, the proposed review will highlight factors explaining *how* or *why* the association between anxiety and alcohol, or alcohol and anxiety, occurs. If modifiable, these mediators can then be targeted in prevention, early intervention, and treatment approaches. To get a complete understanding of the anxiety and alcohol relationship, investigating the role of moderating variables is also necessary. The synthesis of moderators will highlight factors that affect the direction or strength of the relationship between X and Y [27]. As such, moderators can help understand *when* the relationship between anxiety and alcohol or alcohol and anxiety will occur. Given the breadth of possible mediating and moderating mechanisms studies can investigate, the review may identify mechanisms that can act as both a mediator and a moderator in the anxiety-alcohol relationship. An example of such a factor is positive alcohol use expectancies, with previous research identifying it as both a mediator and moderator in the relationships of interest [29-31].

To our knowledge, this will be the first study to systematically identify and synthesize research on mediators and moderators of the comorbid anxiety and alcohol relationship. The outcomes of the review will specifically answer the following questions:

- What mediates the relationship between anxiety and alcohol use?
- What moderates the relationship between anxiety and alcohol use?

## Methods

### Guidelines and Registration

This protocol was written in accordance with the Preferred Reporting Items for Systematic Review and Meta-Analysis Protocols (PRISMA-P) guidelines [32]. The systematic review has been registered in PROSPERO, the International Prospective Register of Systematic Reviews (CRD42023358402).

### Eligibility Criteria

Guided by the population, exposure, outcome (PEO) framework for conducting systematic reviews of association (etiology) [33], the below criteria will be used to determine eligible studies and structure the search strategy.

#### Population

All human populations are of interest for the proposed systematic review, and as such, no limits will be placed on the study population or participant type. Animal studies will be excluded.

#### Exposure and Outcome

Given the bidirectional nature of anxiety and alcohol comorbidity, the proposed systematic review will include studies investigating these concerns in either direction. Therefore, studies where anxiety is the exposure and alcohol use is the outcome, as well as studies where alcohol use is the exposure and anxiety is the outcome, will be included.

A broad classification will be used to define the anxiety exposure and outcome in this review. Anxiety will be conceptualized in line with the Diagnostic and Statistical Manual of Mental Disorders-Fifth Edition (DSM-5), where anxiety disorders are differentiated from obsessive compulsive and related disorders, and trauma and related-stressor disorders [34]. Per the DSM-5, anxiety disorders include generalized anxiety, social anxiety (social phobia), separation anxiety, selective mutism, specific phobia, agoraphobia, and panic disorder. Both studies that assess disorder-level measures or subclinical symptom measures will be included in the review. In addition to these definitions of anxiety, studies reporting broad symptoms of anxiety or anxious traits, such as anxiety sensitivity (eg, Anxiety Sensitivity Index-3), will also be included.

Like anxiety, a broad classification will be used to define the alcohol use exposure or outcome. This review is interested in the full spectrum of alcohol use and related difficulties and will include, but is not limited to, studies reporting frequency or quantity of alcohol consumption; hazardous, binge, or risky drinking; and alcohol use disorders.

Only studies where both the exposure and outcome are measured in the same individual will be included in this review. Studies looking at mediators or moderators between parents and offspring will be excluded.

#### Study Characteristics

Observational studies including cohort, cross-sectional, and longitudinal studies, and secondary analyses of randomized controlled trials reporting quantitative results will be included. Studies only evaluating intervention or treatment outcomes, including mediators or moderators of an intervention, will be excluded. Additionally, studies only reporting prevalence data will be excluded. Studies must be published in a peer-reviewed journal, with reviews, meta-analyses, and information in books, reports, letters, or conference abstracts being excluded from this systematic review. No limits will be placed on publication year or study language.

### Information Sources and Search Strategy

Searches will be conducted in the electronic databases Medline (Ovid), PsycINFO (Ovid), Embase (Ovid), Cochrane Central Register of Controlled Trials (Ovid), Scopus, and Web of Science. Search strategies will be tailored to each database and use a combination of keywords and subject headings or medical subject headings (MeSH) where appropriate. A total of 3 overarching themes of anxiety, alcohol, and mediator or moderator will be used to structure the search, with search terms combined using the Boolean operators “AND” and “OR.” No year or language limits will be imposed on any of the searches. The reference lists of eligible studies will also be hand-searched to find additional studies not picked up in the electronic database searches. Full search strategies for each database are presented in Multimedia Appendix 1.

### Study Selection and Extraction

All identified studies will be imported into the web-based systematic review software Covidence [35], where duplicates will automatically be removed. All titles and abstracts will be screened by a primary reviewer, with a random sample of 50% screened independently by a second reviewer. The full text of potentially eligible studies, identified through screening, will then be reviewed and assessed against the eligibility criteria by both reviewers. Disagreement between reviewers at both stages will be resolved through consultation, and if required, a third reviewer. Cohen κ will be calculated at the screening and full-text stage to determine the interrater agreement between reviewers, with a κ statistic of 0.8 considered a strong level of agreement [36]. The results of the screening and study selection process will be summarized with a PRISMA flow diagram in the final systematic review.

Data will be independently extracted by a primary reviewer using a data extraction spreadsheet in Excel (Microsoft Corp). A second reviewer will check and confirm all extracted data, with any disagreements reconciled through discussion. The following information will be extracted, and, if needed, the corresponding authors of included studies will be contacted for missing information:

- Publication details, including study authors and publication year
- Study characteristics, including study design, location, and sample size
- Sample population characteristics; for example, age, sex, ethnicity, socioeconomic status, and education
- Characteristics of anxiety exposure or outcome; for example, type of anxiety or anxiety disorder (if applicable), measure to assess anxiety, and age of onset
- Characteristics of alcohol use exposure or outcome; for example, frequency or quantity of use, age of initiation, hazardous or binge drinking, and alcohol use disorder
- Relevant information on the mediators and moderators examined, including the statistical analysis approach used; for example, mediator or moderator classification, assessment of mediator or moderator, single or multiple mediators or moderators assessed, whether confounders were controlled for, and overall strength of effect

### Risk of Bias

Studies deemed eligible for inclusion in the systematic review will have their quality and risk of bias assessed independently by 2 reviewers. Disagreements between reviewers will be resolved through discussion and consultation with a third reviewer if required. Appraisal will be done using the Joanna Briggs Institute Critical Appraisal Tools [37]. Both the Checklist for Analytical Cross-Sectional Studies and the Checklist for Cohort Studies will be used to accommodate the different research designs of the included studies. These checklists evaluate the study sample, measurement of exposure and outcome variables, confounding, appropriateness of statistical analysis, and follow-up reporting (cohort checklist only) across 11 questions in the cohort studies checklist and 8 questions in the cross-sectional studies checklist.

### Data Synthesis

#### Overview

A narrative synthesis of individual mediators and moderators from the included studies will be performed, with results grouped in line with the biopsychosocial model [38]. This model encompasses biological factors like sex and age, psychological factors including cognitive and behavioral facets, and social factors, which encompass an individual’s broader environment and the society in which they live. Furthermore, it is likely the relationships between anxiety and alcohol use will change between different groups; therefore, subgroup synthesis is planned for age subgroups (as defined by the World Health Organization), anxiety subgroups (as defined by the DSM-5), alcohol use sub groups broken down by severity (eg, mild, moderate, severe alcohol use disorder), and lastly, population types (eg, college students, veterans, specific cultural groups, etc). The inclusion of these subgroups will be reliant on data within the included studies, and if other key groupings emerge, further sub-group analyses may be conducted. Other information extracted, including publication details, study characteristics, sample population characteristics, and exposure and outcome characteristics, will also be narratively synthesized.

Given the broad inclusion criteria, it is anticipated that there will be high heterogeneity across studies. However, if the available data within the included studies is sufficiently homogenous, a meta-analysis will be conducted, with mediation and moderation analyses synthesized separately. Studies with comparable exposure and outcome measures and comparable mediator and moderator variables will be included in the meta-analysis. The indirect effects of mediation models will be synthesized using the methods defined in Cheung [39]. The synthesis of moderating variables will be dependent on the statistical analysis within individual studies but will include pooled estimates of coefficients.

#### Confidence in Cumulative Evidence

The cumulative strength of evidence from included studies will be assessed using the GRADE framework [40]. This framework enables a conclusion to be drawn on whether there is high, moderate, low, or very low confidence in the findings.

## Results

This systematic review is currently underway. In September 2022, initial database searches identified 7330 unique studies for title and abstract screening. It is anticipated that the review, including data extraction and synthesis, will be completed by December 2023. Upon completion, results will be reported in line with PRISMA and disseminated in a peer-reviewed journal. Before submission, searches will be rerun to capture any additional articles that have been published since the initial database searches.

## Discussion

This paper summarizes the protocol for a systematic review and prospective meta-analysis on mediators and moderators in the co-occurring anxiety and alcohol relationship. The review will incorporate studies from a wide spectrum of anxiety and alcohol classifications, including from anxiety symptoms to anxiety disorders and from alcohol use to alcohol use disorders. The biopsychosocial model will be used to synthesize identified factors to determine the distinct roles of biological, psychological, and social mechanisms.

Existing studies often examine a single mediator or moderator, despite evidence of multiple mechanisms contributing to the alcohol and anxiety relationship [12]. In light of this, this review will be the first to consolidate the literature to date on mediators and moderators driving this common comorbidity. Identified mediators and moderators can then be used as targets to develop and refine prevention, early intervention, and treatment approaches for comorbid anxiety and alcohol use concerns. In some cases, the identified mediating and moderating factors may not be modifiable in interventions; nonetheless, synthesizing this evidence will help advance the theoretical basis underlying this common comorbidity. As this review will focus on both the pathway from anxiety to alcohol use and the pathway from alcohol use to anxiety, findings may suggest different prevention and intervention approaches are needed depending on the temporal sequencing of the two disorders. One limitation, however, of the planned review is the inclusion of cohort and cross-sectional studies, which will limit the ability to draw causal conclusions for all results. The inclusion of these study designs is warranted nonetheless, as preliminary searches indicated most mediation and moderation studies for anxiety and alcohol were not longitudinal.

The findings of this review will offer critical insights into the conceptualization of comorbid anxiety and alcohol use, providing a foundation for researchers, clinicians, and policy makers to enhance outcomes in addressing this complex clinical manifestation. These results have the potential to drive advancements in understanding, prevention, and treatment strategies, thereby positively impacting individuals experiencing anxiety and alcohol use concerns.

## Appendix

Multimedia Appendix 1 Electronic database searches.

## Abbreviations

DSM-5: Diagnostic and Statistical Manual of Mental Disorders-Fifth Edition
GRADE: Grading of Recommendations, Assessment, Development, and Evaluations
MeSH: medical subject headings
PEO: population, exposure, outcome
PRISMA-P: Preferred Reporting Items for Systematic Review and Meta-Analysis Protocols

## Data Availability

Data sharing is not applicable to this article as no data sets were generated or analyzed during this study.
