# Supplementary material for: Mediators and Moderators in the Co-Occurring Anxiety and Alcohol Use Relationship: Protocol for a Systematic Review and Meta-Analysis
Source: JMIR Res Protoc. 2023 Jul 13;12:e48875. doi: 10.2196/48875 (PMC10375399; doi:10.2196/48875)
Supplement: Multimedia Appendix 1 [file resprot_v12i1e48875_app1.docx]

| **Database: Medline (via OVID)** | | |
| --- | --- | --- |
| Anxiety | 1 | *Anxiety/ |
|  | 2 | *anxiety disorders/ or *agoraphobia/ or *panic disorder/ |
|  | 3 | (Anxiety disorder or separation anxiety disorder or selective mutism or SM or specific phobia or SP or social phobia or social anxiety or SA or panic disorder or PD or agoraphobia or generali?ed anxiety disorder or GAD).tw. |
|  | 4 | (Anxiety sensitivity or AS).tw. |
|  | 5 | (Internali*ing disorder or internali*ing).tw. |
|  | 6 | (Social-emotional disorder* or emotional disorder*).tw. |
|  | 7 | 1 or 2 or 3 or 4 or 5 or 6 |
| Alcohol | 8 | exp Alcohol Drinking/ or exp Alcohol-Related Disorders/ |
|  | 9 | ((alcohol* or drink*) adj3 (misuse* or initiat* or abus* or problem or heavy or binge or disorder* or dependen* or frequen*)).tw. |
|  | 10 | 8 or 9 |
| Mediator/Moderator | 11 | (mediat* or moderat*).mp. |
|  | 12 | (associat* or relat* or role*).ti. |
|  | 13 | (Risk factor* or protective factor* or indirect* or direct*).mp. |
|  | 14 | 11 or 12 or 13 |
|  | 15 | 7 and 10 and 14 |

| **Database: APA PsycInfo (via OVID)** | | |
| --- | --- | --- |
| Anxiety | 1 | exp anxiety/ |
|  | 2 | *anxiety disorders/ or *generalized anxiety disorder/ or *panic attack/ or *panic disorder/ or *separation anxiety disorder/ |
|  | 3 | (Anxiety disorder or separation anxiety disorder or selective mutism or SM or specific phobia or SP or social phobia or social anxiety or SA or panic disorder or PD or agoraphobia or generali?ed anxiety disorder or GAD).tw. |
|  | 4 | (Anxiety sensitivity or AS).tw. |
|  | 5 | (Internali*ing disorder or internali*ing).tw. |
|  | 6 | (Social-emotional disorder* or emotional disorder*).tw. |
|  | 7 | 1 or 2 or 3 or 4 or 5 or 6 |
| Alcohol | 8 | exp alcohol drinking patterns/ |
|  | 9 | exp "alcohol use disorder"/ |
|  | 10 | ((alcohol* or drink*) adj3 (misuse* or initiat* or abus* or problem or heavy or binge or disorder* or dependen* or frequen*)).tw. |
|  | 11 | 8 or 9 or 10 |
| Mediator/Moderator | 12 | (mediat* or moderat*).mp. |
|  | 13 | (associat* or relat* or role*).ti. |
|  | 14 | (Risk factor* or protective factor* or indirect* or direct*).mp. |
|  | 15 | 12 or 13 or 14 |
|  | 16 | 7 and 11 and 15 |

| **Database: Embase (via OVID)** | | |
| --- | --- | --- |
| Anxiety | 1 | *anxiety/ or *social anxiety/ |
|  | 2 | *anxiety disorder/ or *generalized anxiety disorder/ or *panic/ or *separation anxiety/ |
|  | 3 | (Anxiety disorder or separation anxiety disorder or selective mutism or SM or specific phobia or SP or social phobia or social anxiety or SA or panic disorder or PD or agoraphobia or generali?ed anxiety disorder or GAD).tw. |
|  | 4 | (Anxiety sensitivity or AS).tw. |
|  | 5 | (Internali*ing disorder or internali*ing).tw. |
|  | 6 | (Social-emotional disorder* or emotional disorder*).tw. |
|  | 7 | 1 or 2 or 3 or 4 or 5 or 6 |
| Alcohol | 8 | exp alcohol abuse/ |
|  | 9 | *alcoholism/ |
|  | 10 | *alcohol consumption/ |
|  | 11 | ((alcohol* or drink*) adj3 (misuse* or initiat* or abus* or problem or heavy or binge or disorder* or dependen* or frequen*)).tw. |
|  | 12 | 8 or 9 or 10 or 11 |
| Mediator/Moderator | 13 | (mediat* or moderat*).mp. |
|  | 14 | (associat* or relat* or role*).ti. |
|  | 15 | (Risk factor* or protective factor* or indirect* or direct*).mp. |
|  | 16 | 13 or 14 or 15 |
|  | 17 | 7 and 12 and 16 |

| **Database: Cochrane Central Register of Controlled trials (CENTRAL) (via OVID)** | | |
| --- | --- | --- |
| Anxiety | 1 | anxiety.mp. |
|  | 2 | (Anxiety disorder or separation anxiety disorder or selective mutism or SM or specific phobia or SP or social phobia or social anxiety or SP or panic disorder or PD or agoraphobia or generali?ed anxiety disorder or GAD).tw. |
|  | 3 | (Anxiety sensitivity or AS).tw. |
|  | 4 | (Internali*ing disorder or internali*ing).tw. |
|  | 5 | (Social-emotional disorder* or emotional disorder*).tw. |
|  | 6 | 1 or 2 or 3 or 4 or 5 |
| Alcohol | 7 | alcohol.mp. |
|  | 8 | ((alcohol* or drink*) adj3 (misuse* or initiat* or abus* or problem or heavy or binge or disorder* or dependen* or frequen*)).tw. |
|  | 9 | 7 or 8 |
| Mediator/Moderator | 10 | (mediat* or moderat*).mp. |
|  | 11 | (associat* or relat* or role*).ti. |
|  | 12 | (Risk factor* or protective factor* or indirect* or direct*).mp. |
|  | 13 | 10 or 11 or 12 |
|  | 14 | 6 and 9 and 13 |

| **Database: Scopus** | | |
| --- | --- | --- |
| Anxiety | 1 | ( TITLE ( anxiety OR anxious ) OR TITLE-ABS-KEY ( "anxiety disorder" OR "separation anxiety disorder" OR "selective mutism" OR "specific phobia" OR "social phobia" OR "social anxiety" OR "panic disorder" OR "agoraphobia" OR "generali?ed anxiety disorder" OR "gad" ) ) |
| Alcohol | 2 | ( TITLE ( alcohol ) ) OR ( TITLE-ABS-KEY ( ( ( alcohol* OR drink* ) W/3 ( misuse* OR initiat* OR abus* OR problem* OR heavy OR binge OR disorder* OR dependen* OR frequen* ) ) ) ) |
| Mediator/Moderator | 3 | ( TITLE-ABS-KEY ( mediat* OR moderat* ) OR TITLE ( associat* OR relat* OR role OR "risk factor" OR "protective factor" OR indirect* OR direct ) ) |
|  |  | 1 AND 2 AND 3 |

| **Database: Web of science (core collection)** | | |
| --- | --- | --- |
| Anxiety | 1 | TI=(anxiety OR anxious) |
|  | 2 | TS=("anxiety disorder" OR "separation anxiety disorder" OR "selective mutism" OR "specific phobia" OR "social phobia" OR "social anxiety" OR "panic disorder" OR "agoraphobia" OR "generali?ed anxiety disorder" OR "gad") |
|  | 3 | #2 OR #1 |
| Alcohol | 4 | TI=(alcohol) |
|  | 5 | TS=(( alcohol* OR drink* ) NEAR/3 ( misuse* OR initiat* OR abus* OR problem* OR heavy OR binge OR disorder* OR dependen* OR frequen* )) |
|  | 6 | #4 OR #5 |
| Mediator/Moderator | 7 | TS=(mediat* OR moderat*) |
|  | 8 | TI=(associat* OR relat* OR role OR "risk factor" OR "protective factor" OR indirect* OR direct) |
|  | 9 | #7 OR #8 |
|  | 10 | #3 AND #6 AND #9 |
